# Toll-like receptor signaling in multiple myeloma cells promotes the expression of pro-survival genes B-cell lymphoma 2 and MYC and modulates the expression of B-cell maturation antigen

**DOI:** 10.3389/fimmu.2024.1393906

**Published:** 2024-06-07

**Authors:** Synne Stokke Tryggestad, Ingrid Aass Roseth, Kristin Roseth Aass, Nadia Elise Helene Ørning, Robin Mjelle, Hanne Hella, Therese Standal

**Affiliations:** ^1^ Department of Clinical and Molecular Medicine, Faculty of Medicine and Health Sciences, Norwegian University of Science and Technology, Trondheim, Norway; ^2^ Department of Pathology, St. Olavs University Hospital, Trondheim, Norway; ^3^ Department of Hematology, St. Olavs University Hospital, Trondheim, Norway

**Keywords:** multiple myeloma, drug resistance, BCMA, TLR, carfilzomib, bortezomib, primary cells, proteasome inhibitors

## Abstract

Infections are common in plasma cell cancer multiple myeloma (MM) due to disease-related immune deficiencies and cancer treatment. Myeloma cells express Toll-like receptors (TLRs), and TLR activation has been shown to induce proliferative and pro-survival signals in cancer cells. MM is a complex and heterogeneous disease, and expression levels of TLRs as well as downstream signaling components are likely to differ between patients. Here, we show that in a large cohort of patients, TLR1, TLR4, TLR6, TLR9, and TLR10 are the most highly expressed in primary CD138^+^ cells. Using an MM cell line expressing TLR4 and TLR9 as a model, we demonstrate that TLR4 and TLR9 activation promoted the expression of well-established pro-survival and oncogenes in MM such as *MYC*, *IRF4*, *NFKB*, and *BCL2*. TLR4 and TLR9 activation inhibited the efficacy of proteasome inhibitors bortezomib and carfilzomib, drugs used in the treatment of MM. Inhibiting the autophagosome–lysosome protein degradation pathway by hydroxychloroquine (HCQ) diminished the protective effect of TLR activation on proteasome inhibitor-induced cytotoxicity. We also found that TLR signaling downregulated the expression of *TNFRSF17*, the gene encoding for B-cell maturation antigen (BCMA). *MYC*, *BCL2*, and *BCL2L1* were upregulated in approximately 50% of primary cells, while the response to TLR signaling in terms of *TNFRSF17* expression was dichotomous, as an equal fraction of patients showed upregulation and downregulation of the gene. While proteasome inhibitors are part of first-line MM treatment, several of the new anti-MM immune therapeutic drugs target BCMA. Thus, TLR activation may render MM cells less responsive to commonly used anti-myeloma drugs.

## Introduction

Multiple myeloma (MM) is a plasma cell cancer characterized by the accumulation of clonal plasma cells in the bone marrow. It is the second most common hematologic malignancy, affecting mostly elderly people with a median age of diagnosis of approximately 70 years (1). The prevalence is increasing due to an aging population (2). In most patients, cancer cells produce large amounts of monoclonal antibodies (paraproteins) that can be detected in the circulation (1). Other clinical symptoms include hypercalcemia, renal failure, anemia, and lytic bone lesions (1). MM is a complex and heterogeneous disease, and patients may be grouped into different molecular subtypes based on genetic aberrations. Common initiating genetic events include translocations of the IgH locus and hyperdiploidy. Subsequently, more genetic alterations, such as MYC structural variants and mutations that activate the RAS or NF-κB pathway, are acquired and drive disease progression/development (1, 3, 4). Interferon regulatory factor 4 (IRF4) is a key transcription factor needed for MM cell survival. MYC and IRF4 form an autoregulatory circuit in multiple myeloma, where they induce each other’s transcription to promote MM cell growth and survival (5, 6).

MM is incurable, but several new treatment options in the last two decades have significantly increased life expectancy for the patients. First-line treatment often includes a combination of drugs of different classes, including proteasome inhibitors (PIs), immunomodulating drugs, monoclonal antibodies targeting CD38, and corticosteroids (1). A monoclonal antibody–drug conjugate targeting B-cell maturation antigen (BCMA) expressed on the surface of MM cells and chimeric antigen receptor (CAR) T-cell therapy targeting BCMA has been approved to treat patients who are refractory to previous treatment strategies (7). Bispecific T-cell engagers (BiTEs) targeting BCMA and CD3 have also shown great promise (8–10).

Infections are a major complication of MM and the underlying cause of many deaths (11–14). Indeed, in a recent multi-cohort study including more than 3,700 myeloma patients, 65% of deaths were attributable to infections (15). The increased risk of infections is due to disease-related immunodeficiencies in both humoral and cellular immunity as well as old age and anti-MM therapy (16–18). Even though infections in MM are linked with poor survival, the impact of infectious agents on myeloma cell aggressiveness has not been thoroughly studied. Toll-like receptors (TLRs) play a crucial role in innate immune recognition of microbes and antimicrobial defense. They recognize microbial patterns known as pathogen-associated molecular patterns (PAMPs) and can also recognize damage-associated molecular patterns (DAMPs), which are endogenous molecules derived from damaged cells (19, 20). We and others have shown that TLRs are expressed by primary myeloma cells and MM cell lines (21–28). Moreover, expression of several TLRs increases from diagnosis to relapse in individual patients (23). Several studies have demonstrated proliferative and pro-survival effects of TLR signaling on MM cells (22, 25–29). Moreover, it has been shown that activation of TLR4 in MM cells can reduce the response to bortezomib (27, 28, 30) and the anthracycline adriamycin (29), while TLR7 and TLR9 activation has been shown to reduce the sensitivity to dexamethasone (25) *in vitro*. In contrast, TLR1/2 signaling has been shown to enhance the efficacy of bortezomib (31). Thus, previous studies have demonstrated both protective and sensitizing effects of TLR signaling on bortezomib treatment. Moreover, whether TLR4 or other TLRs in a similar manner may protect against the irreversible proteasome inhibitor carfilzomib has not been reported.

In this study, we performed gene expression analyses of MM cells exposed to TLR4 and TLR9 agonists. We found that both TLR4 and TLR9 activation induced the expression of well-established pro-survival genes and oncogenes in MM such as *MYC*, *IRF4*, *NFKB*, and *BCL2*. We also demonstrate that TLR4 and TLR9 signaling protects against both reversible and irreversible PIs and that the TLR-induced expression of pro-survival genes was maintained in the presence of the PIs. Inhibiting the autophagosomal degradation pathway by hydroxychloroquine (HCQ) diminished the protective effect of TLR activation on proteasome inhibitor-induced cytotoxicity. We also found that TLR signaling downregulated gene and cell surface expression of the drug target BCMA. Thus, TLR activation may render MM cells less responsive to commonly used anti-myeloma drugs.

## Methods

### TLR expression analyses in the CoMMpass patient dataset

For analyses of TLR expression in primary myeloma cells from patients, RNA sequencing data from CD138^+^ cells (MMRF_CoMMpass_IA14a_E74GTF_Salmon_Gene_Counts) were downloaded from the Multiple Myeloma Research Foundation CoMMpass IA14 release (https://research.themmrf.org/). The raw counts were cpm normalized and log transformed using EdgeR before TLR gene expression in diagnostic samples was analyzed (n = 772 patients). Spearman’s correlation test between TLR expression and chromosomal abnormalities was performed using the function *cor.test* in R, and significance was calculated using the function *cor.mtest* in R.

### Cell culture

The MM cell line RPMI-8226 was obtained from the American Type Culture Collection (ATCC; Rockville, MD, USA). RPMI-8226 cells depleted for TLR4 (TLR4 KO), TLR9 (TLR9 KO), and control/mock (WT) cells were generated by gene editing using CRISPR/Cas9 as previously described (23). The cells were cultured in RPMI-1640 medium with 20% heat-inactivated fetal calf serum (FCS) during expansion and 10% FCS for experiments. Freshly isolated primary myeloma cells were obtained from the local hospital biobank (Biobank1). CD138^+^ cells were isolated from patient-derived bone marrow aspirates using the RoboSep automated cell separator and the Human CD138 Positive Selection Kit (StemCell Technologies, Grenoble, France). For patient characteristics, see **Supplementary Table 1**. The primary cells were cultured in RPMI-1640 with 2% human serum, 1 ng/mL rhIL-6 (Gibco, Thermo Fisher Scientific, Waltham, MA, USA), and 20 µg/mL gentamicin. The study was approved by the regional ethics committee (REK #247909), and all patients gave informed consent before donation. The study was performed in accordance with the Declaration of Helsinki. Primary cells and cell lines were cultured at 37°C in a humidified atmosphere with 5% CO_2_.

### Agonists and inhibitors

The following TLR agonists were used for stimulation of MM cells: TLR2/1, Pam3Cys (EMC Microcollections, Tübingen, Germany); TLR2/6, FSL-1 (EMC Microcollections); TLR3, Poly(I:C) HMW (Invivogen, San Diego, CA, USA); TLR4, Ultrapure LPS (E.coli 0111:B4, Invivogen); TLR5, Flagellin FLA-ST (Invivogen); TLR7 and TLR8, R-848 (Invivogen); and TLR9, CpG ODN 2006 (TIB Molbiol, Berlin, Germany). The following concentrations were used: Pam3Cys, 1 µg/mL; FSL-1, 1 µg/mL; Poly(I:C), 10 µg/mL; LPS, 0.1 µg/mL for RPMI-8226 cells and 1.0 µg/mL for primary MM cells; Flagellin, 1 µg/mL; R-848, 1 µg/mL; and CpG, 1 µM. For controls, cells were treated with equal volumes of cell culture medium. Patient samples with a limited cell number were treated with a cocktail containing all agonists.

For experiments assessing sensitivity to proteasome inhibitors, carfilzomib (#S2853) and bortezomib (#S1013) from Selleck Chemicals (Houston, TX, USA) were used at concentrations as indicated in the figure legends. For inhibition of the autophagy-lysosomal pathway, bafilomycin A1 (#sc-201550A) from Santa Cruz Biotechnology (Dallas, TX, USA) and hydroxychloroquine from Sigma-Aldrich (St. Louis, MO, USA) were used at concentrations as indicated in the figure legends.

### BrdU proliferation assay

Proliferation was assessed by quantification of cells in cell cycle S-phase, measured as 5-bromo-2′-deoxyuridine (BrdU)-positive live cells using flow cytometry. Cells were seeded at a concentration of 250,000 cells/mL and treated with LPS or CpG. The following day, additional media containing TLR agonists was added to ensure exponential growth of the cells. Following 48 hours of TLR stimulation, the cells were incubated with 10 µM BrdU (#ab142567, Abcam, Cambridge, UK) in cell culture media for 4 hours. Cells were washed in phosphate-buffered saline (PBS) and further stained with Fixable Viability Dye eFluor™ 450 (#65–0863-14, eBioscience, San Diego, CA, USA) for 30 minutes on ice. The cells were washed with PBS and fixed with ice-cold methanol. Fixed cells were kept in the fridge in methanol overnight for further analysis. The following day, cells were washed with PBS before DNA was hydrolyzed by slowly adding 1 mL of 2N HCl/0.05% Triton X-100 upon vortexing. Following 30 minutes of incubation at room temperature, 1 mL of 0.1 M Na_2_B_4_O_7_ (borax) was added to neutralize the pH. Cells were spun down and resuspended in PBS with 1% bovine serum albumin (BSA)/0.1% Tween. Next, cells were stained with Alexa Fluor 488 Anti-BrdU (#ab220074, Abcam) for 1 hour at room temperature. Cells were washed before analysis on the LSR II instrument (BD Biosciences, San Jose, CA, USA) with FACS Diva software (BD Biosciences). FlowJo version 10 (TreeStar, Ashland, OR, USA) was used to gate BrdU positive cell fractions of live cells.

### Cell viability assay

Cell viability measurements were performed using CellTiter Glo 2.0 Cell Viability Assay from Promega (Madison, WI, USA) according to the manufacturer’s protocol. Ten thousand cells were seeded per well before treatment with LPS or CpG. For experiments investigating the effect of TLR activation on sensitivity to proteasome inhibitors, cells were pre-treated with LPS or CpG for 2–2.5 hours before treatment with bortezomib/carfilzomib or dimethyl sulfoxide (DMSO) control. For experiments investigating autophagy, cells were pre-treated with LPS or CpG for 4 hours before treatment with bortezomib or carfilzomib and HCQ. Cells were incubated for 24–48 hours before viability was assessed. Luminescence was recorded using a Victor3 plate reader and Wallac 1420 Work Station software (PerkinElmer Inc., Waltham, MA, USA) or the PolarSTAR OMEGA plate reader with MARS Data Analysis Software version 2.10 R3 (BMG Labtech GmbH, Ortenberg, Germany).

### NanoString gene expression analysis

mRNA transcript counts in RPMI-8226 cells challenged with LPS for 48 hours were performed using the nCounter analysis system (NanoString Technologies, Seattle, WA, USA) and the gene expression panels Human Metabolic pathways panel (XT-CSO-HMP1–12) and the PanCancer pathways Panel (XT-CSO-PATH1–1) from NanoString Technologies. The assay was performed according to the manufacturer’s protocol using 100 ng total RNA input. nSolver Analysis Software (NanoString Technologies) was used for the calculation of transcript numbers, applying normalization against housekeeping genes. Using normalized count matrices, differentially expressed genes between control and LPS-stimulated RPMI-8226 cells were analyzed in R using linear models for microarray and RNA-seq data (limma-voom) with TMM normalization. p-Values were adjusted using the Benjamini–Hochberg correction. Analyses were run using R version 4.3.2 (2023–10-31). The packages used with version number include the following: packageVersion (“org.Hs.eg.db”) ‘3.14.0’, packageVersion (“limma”)’3.50.3’, packageVersion (“edgeR”) ‘3.36.0’, and packageVersion (“ggplot2”) ‘3.4.4’. To identify which biological pathways were most affected by LPS stimulation, the significantly differentially expressed genes (adjusted p-value <0.05, N = 112) were used as input in a gene set enrichment analysis (Enrichr) (32) using the Panther metabolic and cell signaling database.

### Seahorse metabolic assays

Oxygen consumption rate (OCR) and extracellular acidification rate (ECAR) were measured using the Seahorse XF96 bioscience extracellular flux analyzer (Agilent, Santa Clara, CA, USA). Seahorse XF cell culture microplates were coated with Cell-Tak (#354240, Corning, New York, NY, USA) following the manufacturer’s protocol, and cells were seeded at a concentration of 25,000 cells/well. Glycolysis and oxidative respiration were measured by combining the Glycolysis stress test and Mito stress test assays (Agilent) to allow for assessment of glycolysis and oxidative respiration in the cells at the same time, as previously described (33). Seahorse XF base medium was supplemented with 10 mM sodium pyruvate and 2 mM glutamine (both from Sigma-Aldrich). Glucose (Merck) at mass of 10 mM was added in the first injection, followed by 1 µM oligomycin in the second injection, 0.8 µM carbonyl cyanide *p*-trifluoro-methoxyphenyl hydrazone (FCCP) (#C2920) in the third injection, and finally, a combination of 2 µM rotenone (#R8875) + 2 µM antimycin (#A0149) + 50 mM 2-deoxyglucose (2-DG; #D8375), all from Sigma-Aldrich. Basal respiration was determined using the following formula: (last rate measurement before oligomycin injection) − (non-mitochondrial respiration rate). Glycolysis was determined using the following formula: (maximum rate measurement before oligomycin injection) − (last rate measurement before glucose injection).

### Real-time quantitative PCR

Primary cells were stimulated with a cocktail of TLR agonists covering TLR1–TLR9 (concentrations as described above) for 6 hours. RNeasy mini kit was used to isolate total RNA (Qiagen, Hilden, Germany) before cDNA was synthesized with a High Capacity RNA-to-cDNA kit (Applied Biosystems, Carlsbad, CA, USA). Real-time quantitative PCR was performed using TaqMan Gene Expression Assays (Thermo Fisher, Waltham, MA, USA) and the StepOnePlus Real-Time PCR system (Applied Biosystems) using standard settings (2 minutes 50°C, 10 minutes 95°C, 40 cycles at 95°C for 15 sec, and 1 minute 60°C). The comparative Ct-method was used to estimate relative changes in gene expression, and *ACTB* (for CD138^+^ primary cells) or *TBP* (for RPMI-8226) were used as housekeeping genes. The following gene expression assays were used: ACTB (Hs01060665_g1), TBP (Hs00427620_m1), IRF4 (Hs01056533_m1), MYC (Hs00153408), BCL2 (Hs00608023_m1), BCL2L1 (Hs00236329_m1), MCL1 (Hs01050896_m1), and TNFRSF17 (Hs03045080_m1).

### Immunoblotting

Cells were lysed in lysis buffer containing 50 mM Tris–HCl, 1% NP40, 150 mM NaCl, 10% glycerol, 1 mM Na_3_VO_4_, 50 mM NaF, and cOmplete Protease Inhibitor (Roche Diagnostics, Mannheim, Germany). To assess the protein expression of SQSTM1, cells were lysed in 8 M urea 0.5% Triton X-100, and lysates were sonicated to shear DNA before electrophoresis. Lysates were mixed with 1× NUPage LDS sample buffer supplemented with 0.1 mM DTT and heated at 70°C for 10 minutes for protein denaturation. Samples were next separated on NuPAGE™ 4–12% Bis-Tris polyacrylamide gel before the proteins were transferred to a nitrocellulose membrane using the iBlot Dry Blotting System (Invitrogen, Thermo Fisher) or by wet blotting using the Criterion™ Blotter (Bio-Rad, Hercules, CA, USA). The membranes were blocked with 5% bovine serum albumin in 0.1% Tris-buffered saline with 0.01% Tween (TBS-T) or 5% dry milk in TBS-T, followed by incubation with primary antibodies for 1–3 days. Horseradish peroxidase (HRP)-conjugated antibodies (DAKO, Glostrup, Denmark) and Super Signal West Femto Maximum Sensitivity Substrate (Thermo Fisher Scientific) were used for detection, and images were obtained using Odyssey Fc Imager (LI-COR Biosciences, Cambridge, UK). Images were analyzed using Image Studio Software (LI-COR Biosciences). The following primary antibodies were used, all from Cell Signaling Technology (Danvers, MA, USA): anti-β-actin (#4967), anti-p-IκBα (#2859), anti-IκBα (#4812), anti-c-Myc (#5605), anti-IRF4 (#4964), anti-Bcl-2 (#15071), anti-Bcl-xl (#2762), and anti-SQSTM1/p62 (#39749).

### Proteasome activity assay

Chymotrypsin-like proteasome activity was measured using Proteasome-Glo™ Chymotrypsin-Like Cell-Based Assay (#G8660, Promega) according to the manufacturer’s protocol. RPMI-8226 cells were seeded at a concentration of 10,000 cells/well. Cells were pre-treated with LPS or CpG for 2 hours before treatment with bortezomib. Following 24 hours, equal numbers of the treated cells were transferred to separate wells for analysis of chymotrypsin-like proteasome activity and cell viability. Cell viability was measured using CellTiter Glo 2.0 Cell viability assay (Promega) according to the manufacturer’s protocol. Luminescence was measured using the PolarSTAR OMEGA plate reader with MARS Data Analysis Software version 2.10 R3 (BMG Labtech GmbH, Ortenberg, Germany). Data are presented as chymotrypsin-like proteasome activity normalized against cell numbers.

### Flow cytometry

BCMA surface expression in RPMI-8226 cells treated with LPS or CpG for 48 hours was analyzed by flow cytometry. TLR-stimulated cells were washed with PBS before being stained with eBioscience™ Fixable Viability Dye eFluor™ 450 (#65–0863-14, Invitrogen) for 30 minutes according to the manufacturer’s protocol. Cells were washed twice in flow buffer (0.1% BSA in PBS) before being stained with APC anti-human CD269 (BCMA) Antibody (#357506, BioLegend, San Diego, CA, USA) for 30 minutes on ice. Cells were washed twice in flow buffer and analyzed using LSR II (BD Biosciences) and FACS Diva software (BD Biosciences). Samples were analyzed using FlowJo V.10.4 (TreeStar, Ashland, Oregon, USA). Debris and dead cells were gated out before the median fluorescence intensity (MFI) of BCMA expression was analyzed. Fluorescence minus one (FMO) controls not stained with APC anti-human CD269 (BCMA) antibody were used as a negative staining control.

### Statistical analyses

Statistical analyses were performed using GraphPad Prism Version 10.2.0 for MacOS (GraphPad Software). For comparison of more than two groups, one-way or two-way ANOVA followed by Sidak’s multiple comparisons test was used as indicated. Differences were considered significant when p < 0.05.

## Results

### Primary myeloma cells express a broad range of TLRs

**Figure 1:**
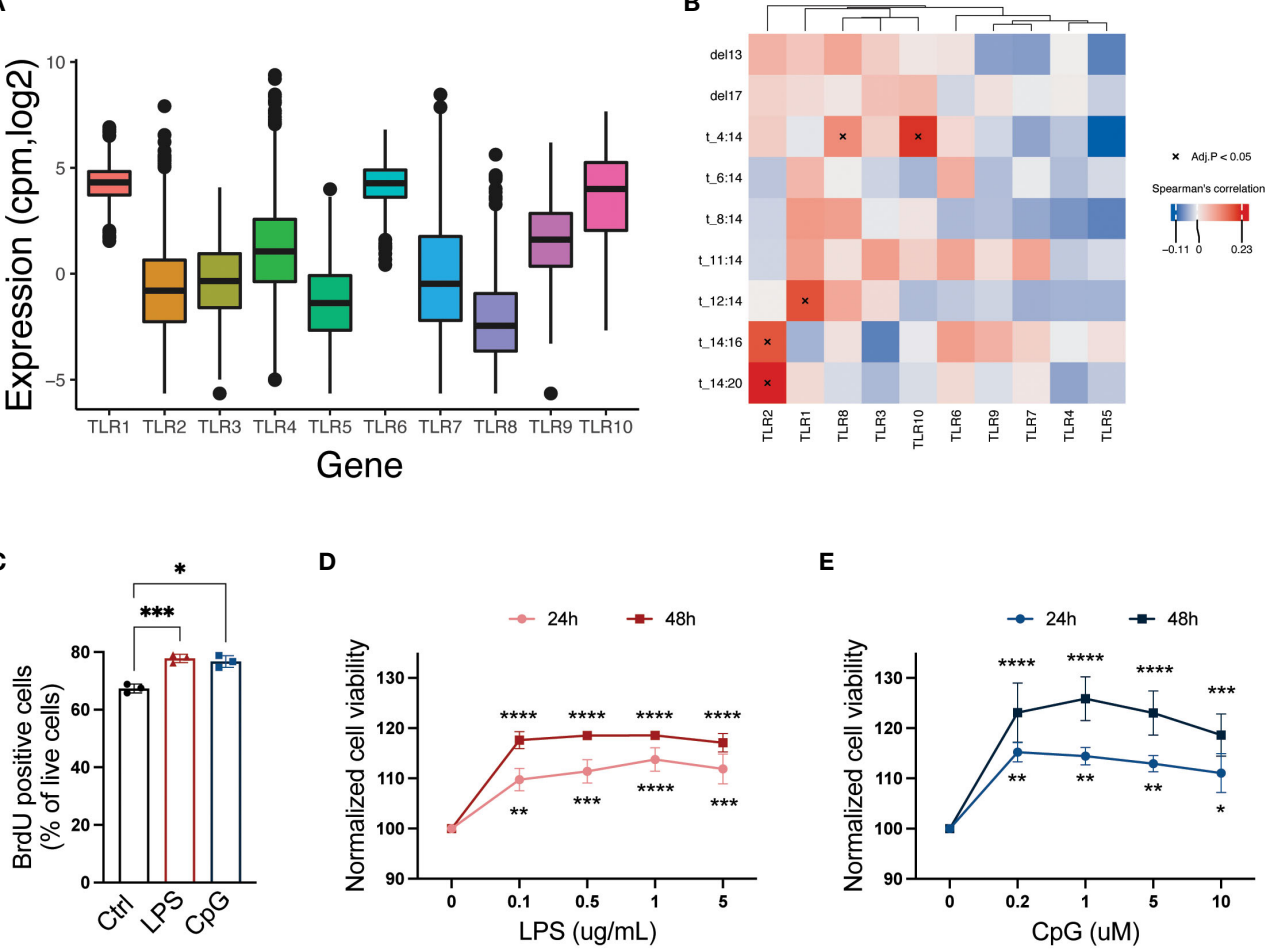
A broad repertoire of TLRs is expressed by CD138^+^ cells isolated from MM patients, and activation of TLRs promotes cell proliferation and survival. **(A)** TLR1–10 mRNA expression in CD138^+^ cells isolated from MM patients (N = 772). Median expression is indicated. Data were obtained from the CoMMpass dataset IA14. **(B)** Heatmap showing correlation between TLR genes and chromosomal abnormalities in MMRF CoMMpass dataset IA14. Spearman’s correlation coefficients are colored, and significant correlations (Benjamini–Hochberg adjusted p-values) are indicated with asterisk. **(C)** Fraction of 5-bromo-2′-deoxyuridine (BrdU)-positive live RPMI-8226 cells upon treatment with LPS (0.1 µg/mL) or CpG (1 µM) for 48 hours. The figure shows mean ± SEM of three independent experiments. p-Values were calculated using one-way ANOVA; *p ≤ 0.05, ***p ≤ 0.001. **(D, E)** RPMI-8226 cells were treated with LPS or CpG at concentrations as indicated for 24 and 48 hours before viability was measured by CellTiter Glo assay. The figure shows mean ± SEM of three independent experiments. p-Values were calculated using two-way ANOVA followed by Sidak’s multiple comparisons test, *p ≤ 0.05, **p ≤ 0.001, ***p ≤ 0.001, ****p ≤ 0.0001. TLRs, Toll-like receptors; MM, multiple myeloma.

To obtain an overview of TLR expression in primary MM cells, we examined TLR mRNA expression in CD138^+^ cells obtained from 772 patients at diagnosis (CoMMpass IA14). We found that overall, TLR1, TLR4, TLR6, TLR9, and TLR10 were the most highly expressed (median cpm > 2) in this cohort. Importantly, however, the expression levels varied considerably between patients (**Figure 1A**). Using data from CoMMpass, we investigated the correlation between the presence of chromosomal abnormalities and gene expression of TLR genes. We found that four TLR genes correlated significantly with chromosomal abnormalities, of which TLR2 correlated with t(14:20) and t(14:16), TLR1 correlated with t(12:14), and TLR8 and TLR10 both correlated with t(4:14) (**Figure 1B**).

### TLR activation promotes MM cell proliferation and viability

The MM cell line RPMI-8226 expresses a broad range of TLRs (23) including TLR4 and TLR9, which were also expressed in a high proportion of the primary MM cells. In line with previously published data (22, 25, 27–29), we found that both cell proliferation (**Figure 1C**) and cell viability (**Figures 1D, E**) were increased following LPS and CpG treatment.

### TLR activation induces expression of pro-survival and anti-apoptotic factors

**Figure 2:**
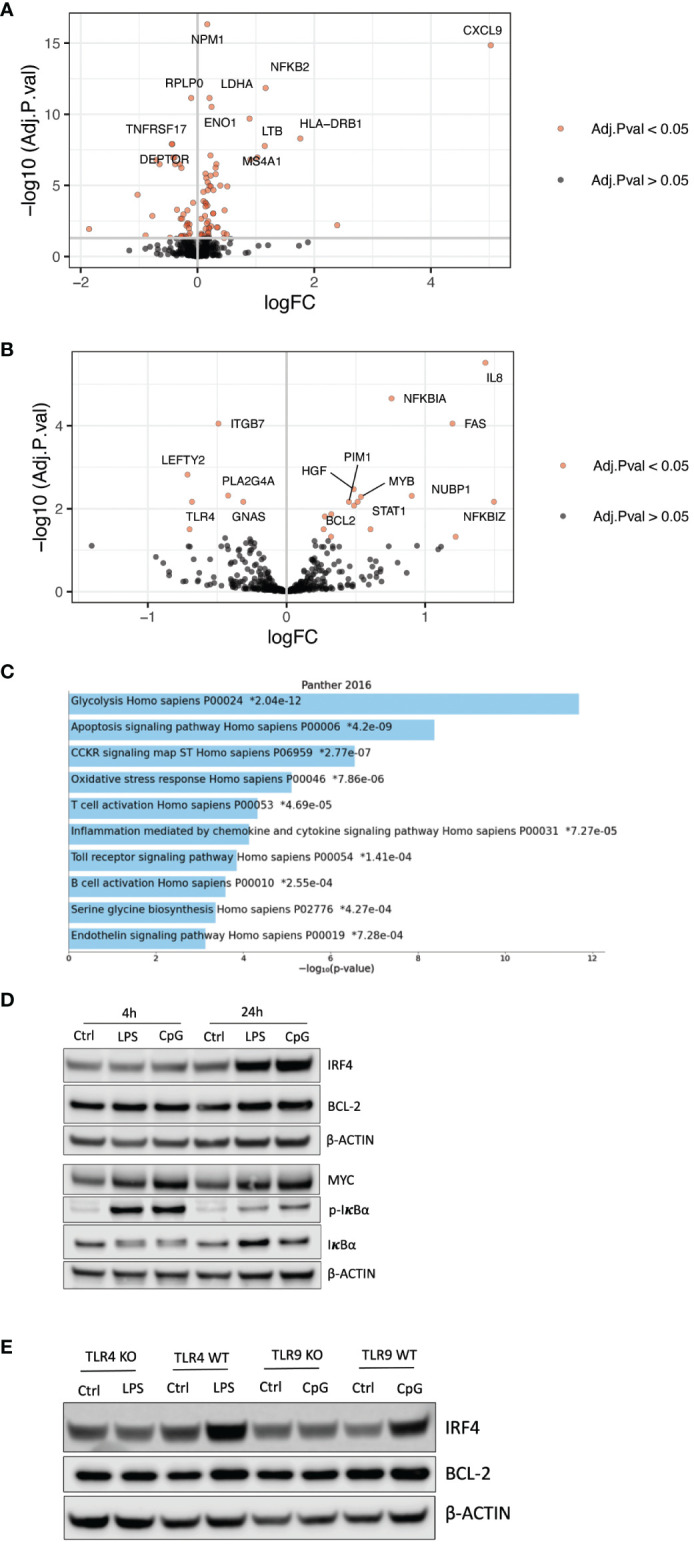
TLR activation induces pro-survival and anti-apoptotic factors. **(A)** Volcano plot showing genes that were significantly differently expressed (adjusted p-value <0.05) in RPMI-8226 cells treated with LPS (0.1 µg/mL) for 48 hours as determined by mRNA transcript counting using the nCounter Human Metabolic pathways panel. Genes with logFC > 0 or logFC < 0 and −log10 (adj. p-value) >7.5 were annotated. Significance was determined using limma-voom with TMM normalization and adjusted using Benjamini–Hochberg correction. **(B)** Volcano plot showing genes that were significantly differently expressed (adjusted p-value <0.05) in RPMI-8226 cells treated with LPS (0.1 µg/mL) for 48 hours determined by mRNA transcript counting using the nCounter Human PanCancer pathways panel. Genes with logFC > 0 or logFC < 0 and −log10 (adj. p-value) >2 were annotated. Significance was determined using limma-voom with TMM normalization and adjusted using Benjamini–Hochberg correction. **(C)** Gene set enrichment analysis of significantly differentially expressed genes (adj. p < 0.05) in RPMI-8266 cells stimulated with LPS (0.1 µg/mL) compared with control cells. The top significantly enriched biological processes shown are ordered by the adjusted p-values. **(D)** Immunoblot of RPMI-8226 cells treated with LPS (0.1 µg/mL) and CpG (1 µM) for 4 and 24 hours. **(E)** Immunoblot of TLR4 KO and WT and TLR9 KO and WT cells treated with LPS (0.1 µg/mL) or CpG (1 µM) for 24 hours, respectively. The blot is representative of three biological replicates. TLR, Toll-like receptor.

To characterize how TLR4 activation altered the cells’ gene expression, we examined mRNA expression in RPMI-8226 cells after LPS treatment for 48 hours using NanoString gene expression assays. For genes present in the Human Metabolic pathways panel, 57 were significantly increased, and 34 were downregulated upon LPS stimulation (**Figure 2A**; **Supplementary Table 2**). For the PanCancer pathways panel, a total of 16 genes were significantly upregulated with LPS treatment, and six genes were downregulated (**Figure 2B**; **Supplementary Table 3**). Genes that were significantly differently expressed between LPS-stimulated and unstimulated cells were enriched in pathways such as glycolysis, apoptosis, and oxidative stress response (**Figure 2C**). In support of these findings, we found that TLR signaling increased glycolysis and basal OXPHOS (**Supplementary Figures 1A, B**). Importantly, among the upregulated genes, there were several genes encoding established pro-survival and anti-apoptotic factors in myeloma, including *MYC*, *IRF4*, *NFKB1/2*, *BCL2*, and *BCL2L1* (**Supplementary Tables 2, 3**). Gene expression analyses by qPCR demonstrated that also TLR9 signaling in RPMI-8226 cells induced similar changes in terms of anti-apoptotic/pro-survival genes (**Supplementary Figure 1C**). MYC, IRF4, NFKB, and BCL2 were further upregulated at the protein level in response to LPS and CpG (**Figure 2D**). Moreover, cells depleted of TLR4 (TLR4 KO) and TLR9 (TLR9 KO) did not change their protein expression of IRF4 and BCL2 in response to TLR stimulation, supporting that the effect is dependent on TLR signaling (**Figure 2E**). Another striking effect of TLR stimulation was the increase in several proteasomal components as well as an increase in the expression of the SQSTM1, an autophagosomal cargo receptor (**Supplementary Table 2**). Thus, TLR stimulation may promote an increased capacity of the two major protein degradation systems in the cells, i.e., both enhanced degradation through the proteasome and the autophagosome–lysosome pathway.

### TLR activation reduces MM cell sensitivity to proteasome inhibitors

**Figure 3:**
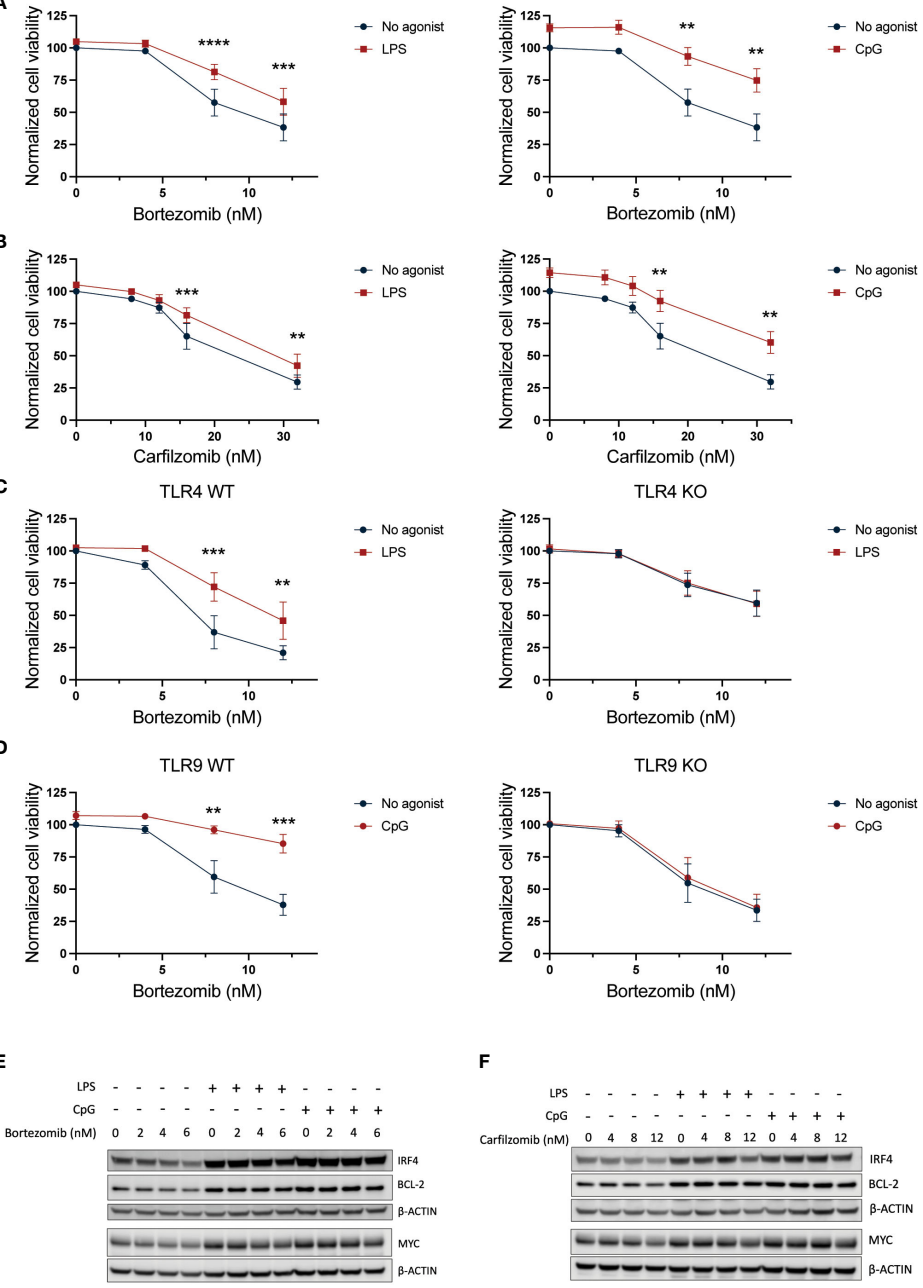
TLR4 and TLR9 activation protects against PI-induced cytotoxicity. **(A, B)** RPMI-8226 cells were treated with LPS (final concentration 0.1 µg/mL) or CpG (final concentration 1 µM) for 2.5 hours before addition of **(A)** 4–12 nM bortezomib and **(B)** 8–32 nM carfilzomib to final concentrations as indicated. Cell viability was measured by CellTiter Glo assay after 24 hours. The figure shows mean ± SEM of three independent experiments. p-Values were calculated using two-way ANOVA and Sidak’s multiple comparisons test. **p ≤ 0.001, ***p ≤ 0.001, ****p ≤ 0.0001. **(C)** TLR4 KO or **(D)** TLR9 KO cells and their respective mock cells (WT) were treated with LPS (0.1 µg/mL) or CpG (1 µM), respectively, for 2.5 hours before addition of 4–12 nM bortezomib. Cell viability was measured by CellTiter Glo assay after 24 hours. The figure shows mean ± SEM of three independent experiments. p-Values were calculated using two-way ANOVA followed by Sidak’s multiple comparisons test. **p ≤ 0.001, ***p ≤ 0.001. **(E)** Immunoblot of RPMI-8226 cells treated with LPS (0.1 µg/mL) or CpG (1 µM) for 2.5 hours followed by treatment with bortezomib at final concentrations as indicated for 24 h. The blot is representative of three independent experiments. **(F)** Immunoblot of RPMI-8226 cells treated with LPS (0.1 µg/mL) or CpG (1 µM) for 2.5 hours followed by treatment with carfilzomib at final concentrations as indicated for 24 h. PI, proteasome inhibitor.

The increase in pro-survival genes combined with a potentially enhanced protein degradation capacity supports reduced efficacy toward proteasomal inhibitors upon TLR signaling. Indeed, both TLR4 and TLR9 activation significantly reduced MM cell sensitivity to both bortezomib and carfilzomib (**Figures 3A, B**). The reduction in drug sensitivity was dependent on TLR signaling since the protective effect of TLR activation was lost in TLR4 and TLR9 KO cells (**Figures 3C, D**; **Supplementary Figures 2A, B**). Importantly, while bortezomib and carfilzomib reduced IRF4, MYC, and BCL2 levels, the expression of these proteins was maintained in cells co-treated with LPS or CpG except for the highest drug concentrations (**Figures 3E, F**), further supporting that TLR-induced expression of these pro-survival factors could be implicated in the reduced drug sensitivity in the presence of TLR agonists.

### Targeting autophagy reduced the effect of TLR activation on PI sensitivity

**Figure 4:**
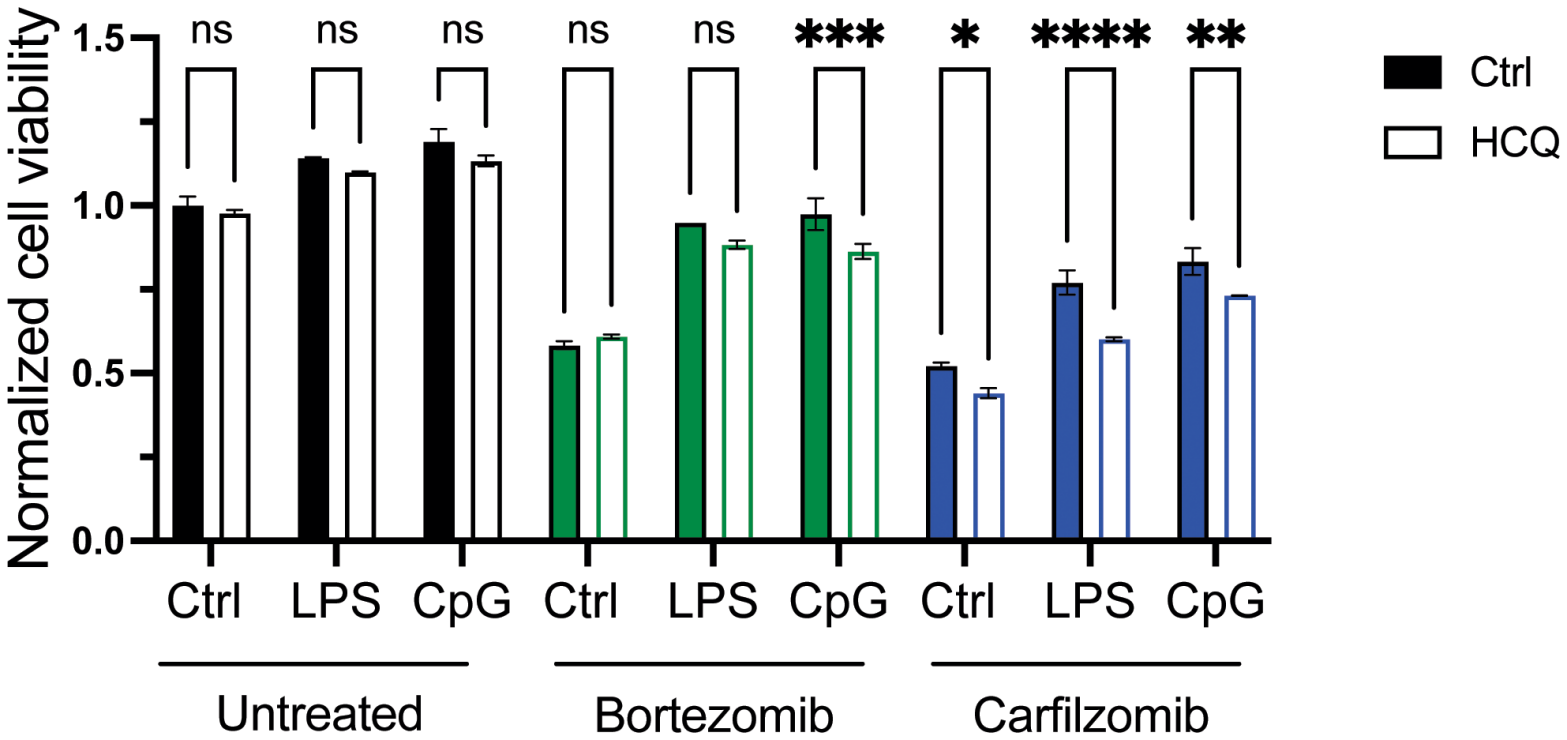
Targeting autophagy reduces the effect of TLR activation on PI sensitivity. RPMI-8266 cells were treated with LPS or CpG for 4 hours, before treatment with DMSO as control (untreated), bortezomib, or carfilzomib in the presence or absence of hydroxychloroquine. Cell viability was assessed by CellTiter Glo assay after 24 hours, and data are presented as normalized values with control cells as a reference. Final concentrations: LPS 0.1 µg/mL, CpG 1 µM, bortezomib 6 nM, carfilzomib 12 nM, and HCQ 10 µM. The figure shows mean ± SD for two technical replicates. p-Values were calculated using two-way ANOVA and Sidak’s multiple comparisons test, *p ≤ 0.05, **p ≤ 0.001, ***p ≤ 0.001, ****p ≤ 0.0001, P > 0.5 = not significant (ns). TLR, Toll-like receptor; PI, proteasome inhibitor; DMSO, dimethyl sulfoxide; HCQ, hydroxychloroquine.

Proteasome subunits were among the upregulated genes in LPS-stimulated MM cells (**Supplementary Table 2**), but TLR activation did not counteract the effect of bortezomib on proteasomal degradation efficacy (**Supplementary Figure 3A**). However, targeting autophagy by adding HCQ slightly but significantly reduced the protective effect of TLR signaling on PI-induced cytotoxicity (**Figure 4**). HCQ has previously been shown to increase the efficacy of carfilzomib, but not bortezomib (34). Indeed, HCQ had no effect on cell survival in cells that were not exposed to PI (untreated) or that were exposed to bortezomib alone (bortezomib, ctrl) but inhibited the effect of carfilzomib (carfilzomib, ctrl). Importantly, HCQ reduced the protective effect of LPS and CpG on both bortezomib-induced cytotoxicity (bortezomib and CpG) and carfilzomib-induced cytotoxicity (carfilzomib, LPS, and CpG). Cells resistant to carfilzomib showed elevated levels of SQSTM1 (34, 35). Here, we also observed increased mRNA levels of SQSTM1 in cells treated with LPS (**Supplementary Table 2**). Moreover, treating cells with LPS or CpG led to the accumulation of SQSTM1 protein, and the increase was also evident upon inhibition of autophagy (**Supplementary Figure 3B**), supporting that TLR activation can increase flux through the lysosomal protein degradation pathway.

### TLR stimulation promotes the expression of *BCL2*, *BCL2L1*, and *MYC* in a large proportion of patient-derived CD138^+^ cells

**Figure 5:**
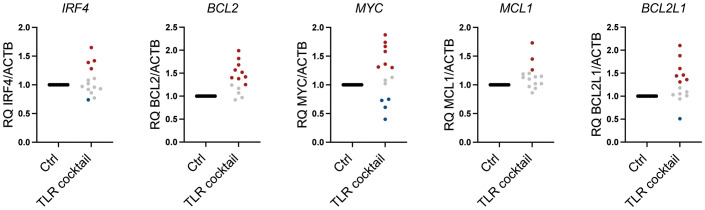
Heterogeneous response to TLR agonists in MM patients. Changes in mRNA expression of *IRF4*, *BCL2*, *MYC*, *MCL1*, and *BCL2L1* (*BCL-XL*) relative to *ACTB* in freshly obtained primary bone marrow CD138^+^ MM cells from 14 donors treated with a cocktail of TLR agonists for 6 hours as determined by RT-qPCR. Each dot represents cells from one individual patient. TLR cocktail: Pam3Cys 1 µg/mL, FSL-1 1 µg/mL, Poly(I:C) 10 µg/mL, LPS 1.0 µg/mL, Flagellin 1 µg/mL, R-8481 µg/mL, and CpG 1 µM. An increase of>25% in relative gene expression is indicated by red dots, while a <25% decrease in relative gene expression is indicated by blue dots. Gray dots indicate less than 25% change in relative mRNA expression. TLR, Toll-like receptor; MM, multiple myeloma.

As shown in **Figure 1A**, plasma cells from patients expressed a broad range of TLRs, but the expression levels varied greatly between patients. Thus, to examine how TLR activation affected pro-survival genes in freshly obtained CD138^+^ cells from MM patients, we treated the cells with a cocktail of TLR agonists covering TLR1–TLR9 for 6 hours. As expected, there were donor variations. However, TLR stimulation increased *BCL2*, *BCL2L1*, and *MYC* in a large proportion of the patients, while the effect on *IRF4* and *MCL1* gene expression was less prominent (**Table 1**; **Figure 5**).

**Table 1:** Changes in gene expression in bone marrow CD138^+^ cells isolated from MM patients stimulated with TLR agonist mix for 6 hours.

	*IRF4*	*BCL2*	*MYC*	*BCL2L1*	*MCL1*	*TNFRSF17*
>25% increase	4/14	9/14	7/14	7/14	3/14	6/14
<25% decrease	1/14	0/14	4/14	1/14	0/14	6/14

MM, multiple myeloma; TLR, Toll-like receptor.

### TLR signaling affects MM cell expression of BCMA

**Figure 6:**
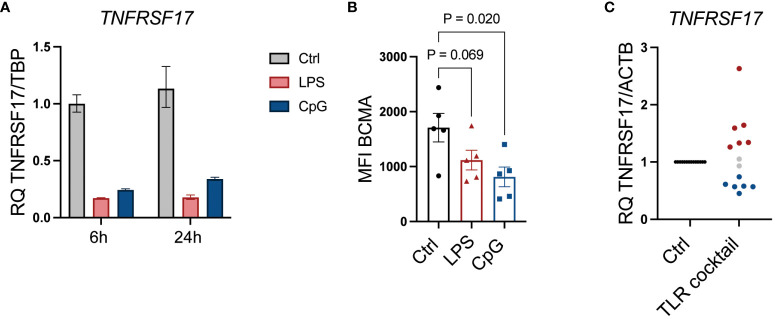
TLR signaling affects MM cell expression of BCMA. **(A)** *TNFRSF17* mRNA expression relative to *TBP* was assessed by RT-qPCR in RPMI-8266 cells treated with LPS (0.1 µg/mL) and CpG (1 µM) for 6 and 24 hours. The figure shows mean of technical replicates. **(B)** Cell surface expression of BCMA (TNFRSF17) was assessed by flow cytometry in RPMI-8266 cells treated with LPS (0.1 µg/mL) or CpG (1 µM) for 48 hours. MFI, median fluorescence intensity. p-Values were calculated using one-way ANOVA and Sidak’s multiple comparisons test. The figure shows mean ± SEM of five independent experiments. **(C)** mRNA expression of *TNFRSF17* relative to *ACTB* in freshly obtained primary bone marrow CD138^+^ MM cells from 14 donors treated with a cocktail of TLR agonists for 6 hours as determined by RT-qPCR. Each dot represents cells from one individual patient. TLR cocktail: Pam3Cys, 1 µg/mL; FSL-1, 1 µg/mL; Poly(I:C), 10 µg/mL; LPS, 1.0 µg/mL; Flagellin, 1 µg/mL; R-848, 1 µg/mL; CpG, 1 µM. An increase of >25% in relative gene expression is indicated by red dots, while a <25% decrease in relative gene expression is indicated by blue dots. Gray dots indicate less than 25% change in relative mRNA expression. TLR, Toll-like receptor; MM, multiple myeloma; BCMA, B-cell maturation antigen.

BCMA is a target for CAR T cells and bispecific antibodies approved to treat MM patients (36). From our NanoString analyses, we observed a significant reduction in *TNFRSF17*, the gene encoding BCMA, upon LPS stimulation (**Figure 2A**; **Supplementary Table 2**). CpG stimulation similarly reduced *TNFRSF17* gene expression (**Figure 6A**), and importantly, cell surface expression of BCMA was also reduced upon TLR4 or TLR9 activation (**Figure 6B**). We also observed the downregulation of *TNFRSF17* in primary cells obtained from 6/14 patients. Interestingly, however, in an equal fraction of the patient samples, *TNFRSF17* was upregulated (**Figure 6C**; **Table 1**). Thus, TLR signaling in MM cells can theoretically both enhance and reduce the efficacy of BCMA-targeting drugs.

## Discussion

The main finding of this work is that TLR9 and TLR4 activation increases the expression of pro-survival genes, provides resistance to both reversible and irreversible proteasome inhibitors, and, at the same time, reduces the expression of BCMA, a broadly expressed surface protein and a target for bispecific antibody therapy and CAR T cells in MM. Thus, infectious agents or ligands released during endogenous stress or damage may act on the MM cells through their TLRs so that they become less responsive to two major drug classes. We also show that TLR expression in MM cells varies greatly between patients and that the response of primary MM cells to TLR agonists is all but uniform.

Proteasome inhibitors are efficient drugs in MM. Their main mode of action is to inhibit proteasomal protein degradation, which leads to the accumulation of misfolded proteins, activation of the unfolded protein response (UPR), and subsequent apoptosis. A cytotoxic effect has also been proposed to be partly due to reduced NF-κB signaling and reduced degradation of pro-apoptotic BCL-2 family members (37, 38). Most patients treated with PIs develop resistance. Reduced sensitivity to PI can be caused by several mechanisms. Proteotoxic stress can be alleviated by increased capacity of the proteasome, e.g., by increased expression of proteasomal subunits (39) or by a compensatory increase in the autophagosome–lysosome protein degradation pathway (35, 37). Resistance to PI has further been associated with de-differentiation of MM cells into cells with a more immature plasma cell profile, with lower production of immunoglobulins and thereby reduced proteasomal load (40). Resistant cells have also been shown to have high mitochondrial metabolic function and increased antioxidant capacity (41–43). Interestingly, here, we found that TLR signaling in MM cells can potentially reduce the sensitivity to PI inhibitors by several of these mechanisms we observed: 1) increased expression of proteasomal components, 2) increased expression of the autophagy cargo receptor SQSTM1, 3) increased expression of pro-survival BCL2-family members, and 4) increased basal glycolysis and OXPHOS.

Autophagy compensates for proteasome insufficiency by clearing protein aggregates (44). Inhibiting the autophagosome–lysosome pathway has been shown to potentiate the effect of bortezomib and carfilzomib in MM cells (34, 35), and targeting autophagy was tolerated and showed promising results in pre-clinical and clinical phase I/II studies (45, 46). Here, we show that inhibiting the lysosomal degradation pathway by HCQ treatment had a potentiating effect on carfilzomib-induced cytotoxicity and that HCQ treatment reduced the protective effect of LPS and CpG stimulation on PI-induced cytotoxicity. Thus, combined targeting of autophagy and proteasomal degradation may be beneficial in patients expressing high levels of TLRs and with an inflammatory or infectious tumor microenvironment.

The pro-survival members of the BCL-2 family, BCL-2, BCL-X_L_, and MCL-1 are frequently upregulated in MM and promote cell survival and drug resistance (47). Importantly, we found that TLR activation upregulated *BCL2* and *BCL2L1* in primary cells from a large fraction of patients. Increased *BCL2* and *BCL2L1* in response to TLR activation may make the MM cells less prone to apoptosis in general, but this was not explored in the current study. Venetoclax is an oral BH3 mimetic that displaces proapoptotic proteins from BCL-2 (48). Venetoclax has shown promising results in combination with bortezomib in patients with t(11;14) translocations and/or high BCL-2 expression (49–51). Strikingly, we also found that MYC was increased in response to TLR activation in 50% of patient samples. MYC is key for MM cell survival and disease development (1, 3–5). Thus, by inducing the expression of key oncogenes and survival genes in MM, TLR activation may make MM cells more resilient to several types of stress and apoptotic signals.

Proteasome inhibitors are the backbone of the most used drug combinations in MM treatment, but new drug classes have shown great efficacy. Several of the new immune therapeutic drugs approved to treat relapsed or refractory MM are targeting BCMA on the myeloma cells (36). Bispecific T-cell engagers with specificity for BCMA and CD3, as well as BCMA-specific CAR T, are highly efficient. All plasma cells express BCMA, and evasion mechanisms include shedding of membrane-bound BCMA as well as antigen loss due to biallelic or monoallelic deletions or mutations in the extracellular domain (52). We show here that TLR4 and TLR9 signaling downregulated BCMA in MM cells at both the gene and protein levels and that approximately 50% of patients’ myeloma cells also respond to TLR activation by downregulating BCMA. In some patients, however, TLR activation promoted the expression of BCMA and may thus enhance the efficacy of drugs targeting BCMA. This contrasting finding may reflect the heterogeneity of TLR expression of myeloma cells as shown here, and the large diversity in genetic alterations in myeloma that may affect downstream signaling pathways. Others have previously shown that TLR activation may be involved in myeloma immune evasion by inducing expression of PD-L1 on cancer cells (29, 53). Thus, TLR signaling may interfere with the efficacy of immune therapy in a broader sense.

In this paper, we studied the effect of TLR signaling on myeloma cells and found that it protected against both bortezomib- and carfilzomib-induced cytotoxicity. Our results are in line with previous studies that demonstrated that TLR4 signaling could protect against bortezomib-induced cytotoxicity by reducing CHOP activity and UPR-mediated apoptosis (27) and by increasing mitochondrial fitness (28, 30). The situation *in vivo* is however far more complex. TLR agonists have been tested as adjuvants to increase the efficacy of cancer vaccines or cancer treatment (54). In MM, it was shown that a TLR9 agonist could restore the ability of patient-derived plasmacytoid dendritic cells (pDCs) to stimulate T-cell proliferation and that activating TLR9 in a xenograft MM model reduced tumor load (55). Whether activation of TLRs in cells present in the tumor microenvironment leads to an overall beneficial effect on tumor control or promotes tumor progression is hard to predict, but the outcome probably depends on which TLRs are expressed on the tumor cells, their genetic makeup, and the status of the immune cells.

Myeloma patients suffer from frequent infections, and their cancer cells may express a broad range of TLRs. Here, we showed that TLR signaling potently promotes the expression of key survival genes *BCL2*, *BCL2L1*, and *MYC* and downregulates the drug target BCMA in a subgroup of patients. In conclusion, our results support that infections may not only be detrimental to patient health but also provide a survival benefit for cancer cells, making them less responsive to proteasome inhibition and to drugs targeting BCMA.

## Data availability statement

The original contributions presented in the study are included in the article/**Supplementary Material**. Further inquiries can be directed to the corresponding author.

## Ethics statement

The studies involving humans were approved by Regional Ethics Committee in Central Norway. The studies were conducted in accordance with the local legislation and institutional requirements. The participants provided their written informed consent to participate in this study.

## Author contributions

ST: Investigation, Writing – original draft, Writing – review & editing, Data curation, Formal analysis, Methodology, Visualization. IR: Investigation, Writing – review & editing. KA: Investigation, Writing – review & editing. NØ: Investigation, Writing – review & editing. RM: Investigation, Writing – review & editing. HH: Writing – review & editing, Methodology. TS: Writing – review & editing, Conceptualization, Funding acquisition, Investigation, Resources, Supervision, Writing – original draft.

## Supplementary material

Supplementary Figure 1
**(A)** Graphical presentation of extracellular acidification rate (ECAR) as a measure of glycolysis in RPMI-8226 cells treated with LPS (0.1 μg/mL) or CpG (1 μM) for 48 hours. The graph shows mean ± SD of more than 8 technical replicates from a representative experiment. Bar graph shows basal glycolysis (ECAR) ± SEM of 3 independent experiments. P-values were calculated using RM One-way ANOVA. *P≤ 0.05. **(B)** Graphical presentation of oxidative consumption rate (OCR) as a measure of oxidative phosphorylation in RPMI-8226 cells treated with LPS (0.1 μg/mL) or CpG (1 μM) for 48 hours. The graph shows mean ± SD of more than 8 technical replicates from a representative experiment. Bar graph shows basal respiration (OCR) ± SEM of 3 independent experiments. P-values were calculated using RM One-way ANOVA. *P≤ 0.05. **(C)** Relative mRNA expression of IRF4 and BCL2 was evaluated by RT-qPCR in RPMI-8266 cells treated with LPS (0.1 μg/mL) or CpG (1 μM).

Supplementary Figure 2
**(A)** TLR4 KO cells and their respective mock cells (WT) were treated with LPS for 2.5 hours before addition of carfilzomib as indicated. Cell viability was measured by CellTiter Glo assay after 24 hours. The figure shows mean ± SEM of 3 independent experiments. P-values were calculated using Two-way ANOVA followed by Sidak’s multiple comparisons test. **p ≤ 0.001, ****p ≤ 0.0001. **(B)** TLR9 KO cells and their respective mock cells (WT) were treated with CpG for 2.5 hours before addition of carfilzomib as indicated. Cell viability was measured by CellTiter Glo assay after 24 hours. The figure shows mean ± SEM of 3 independent experiments. P values were calculated using Two-way ANOVA followed by Sidak’s multiple comparisons test. *p≤ 0.05.

Supplementary Figure 3
**(A)** RPMI-8226 cells were treated with LPS (final concentration 0.1 μg/mL) or CpG (final concentration 1 μM) for 2 hours, before addition of bortezomib (final concentration 2 and 4 nM). Proteasome activity was measured after 24 hours and was normalized against cell viability and against the control. The figure shows mean ± SEM of 4 or 5 independent experiments. P-values were calculated using Two-way ANOVA followed by Sidak’s multiple comparisons test. P>0.5 = not significant (ns). **(B)** Immunoblot of RPMI-8226 cells treated with Bafilomycin to inhibit autophagy (90 μM) for 30 minutes, before treatment with either LPS (0.1 μg/mL) or CpG (1 μM) for 2 hours, before adding bortezomib (4 nM) for 24 hours..

Supplementary Figure 4
**(A)** Gating strategy showing how debris and dead cells were removed before analysis of MFI of BCMA expression. The lower plot shows BCMA expression for a representative experiment.
