# Supplementary figures and images for: Toll-like receptor signaling in multiple myeloma cells promotes the expression of pro-survival genes B-cell lymphoma 2 and MYC and modulates the expression of B-cell maturation antigen
Source: Front Immunol. 2024 Jun 7;15:1393906. doi: 10.3389/fimmu.2024.1393906 (PMC11190062; doi:10.3389/fimmu.2024.1393906)

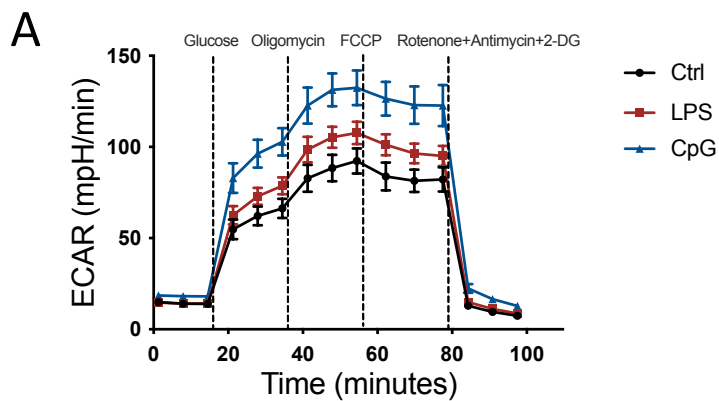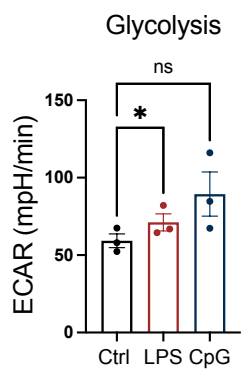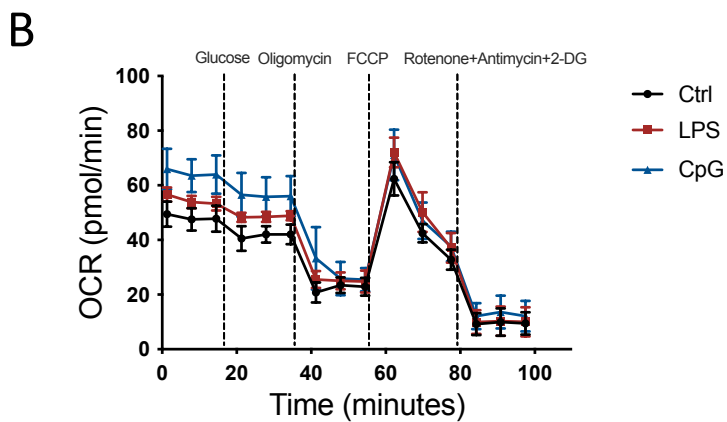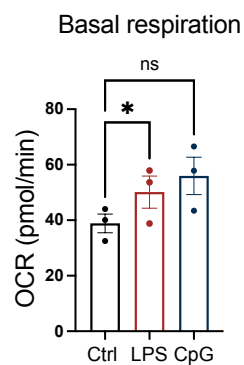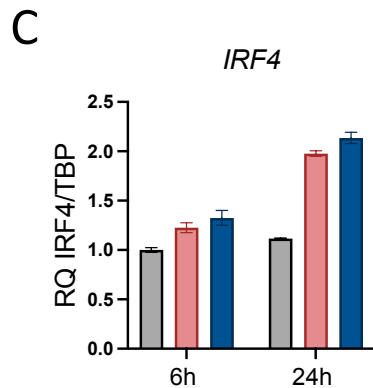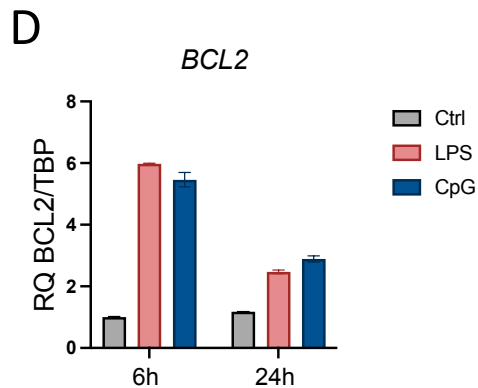

Supplementary Figure 1

A

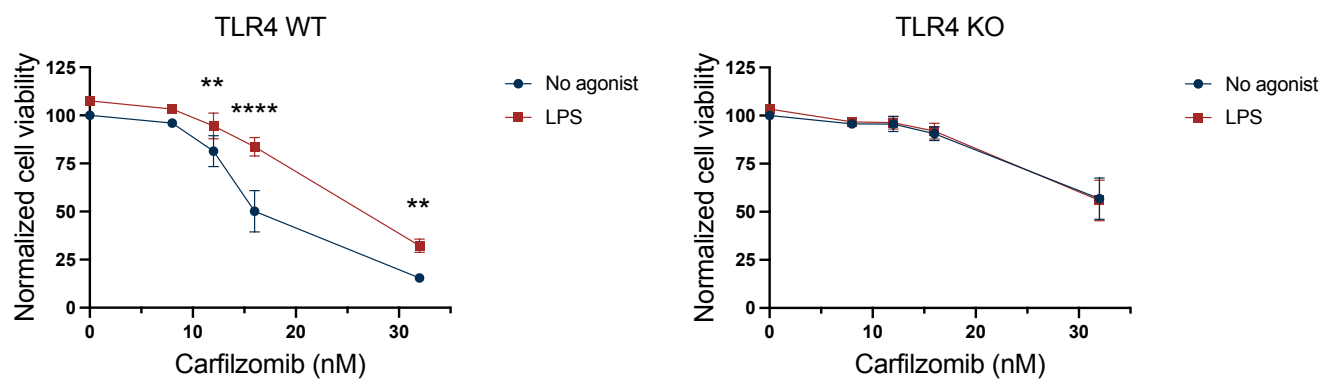

B

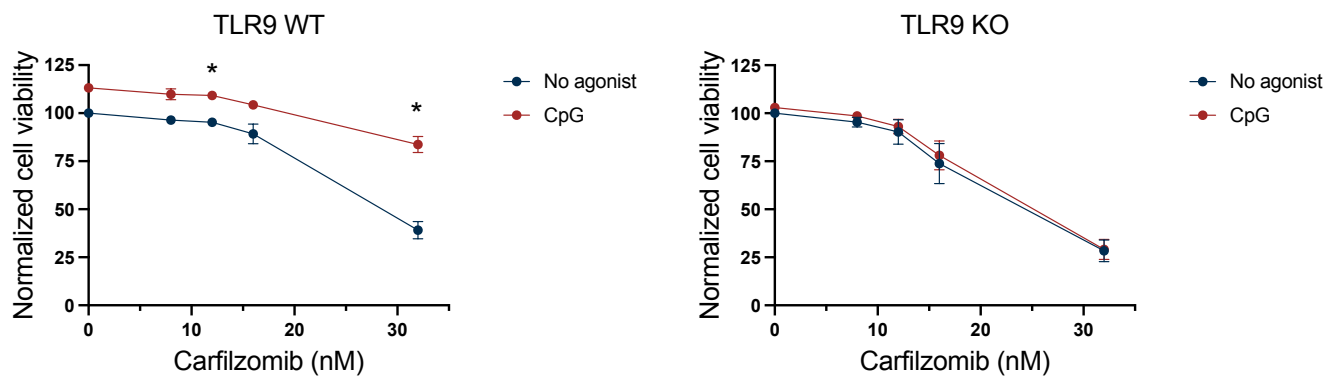

Supplementary Figure 2

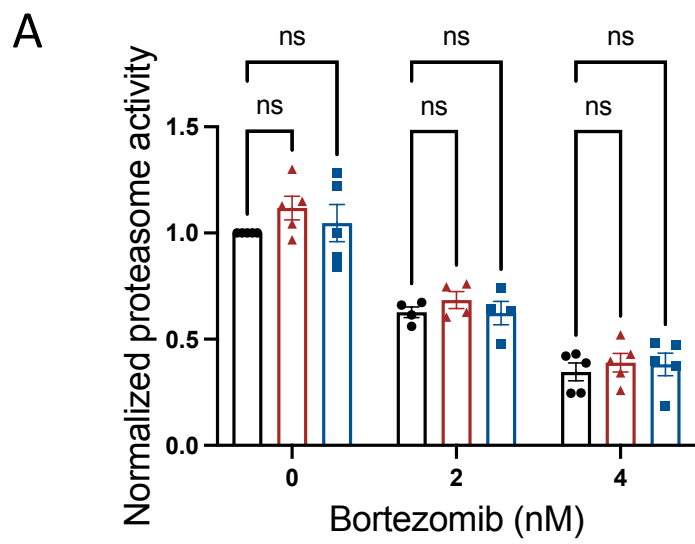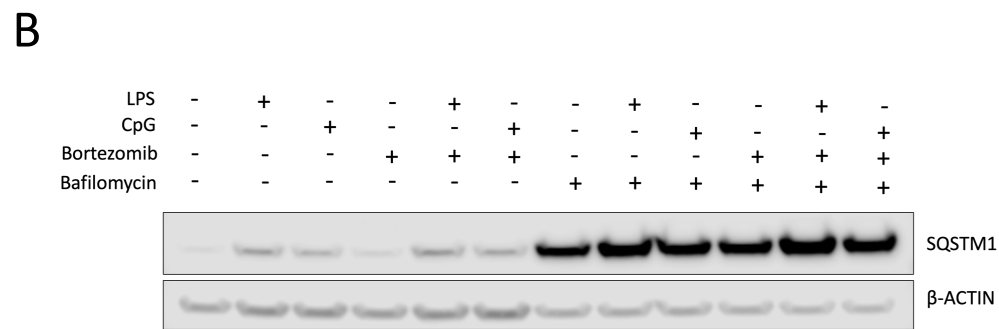

Supplementary Figure 3

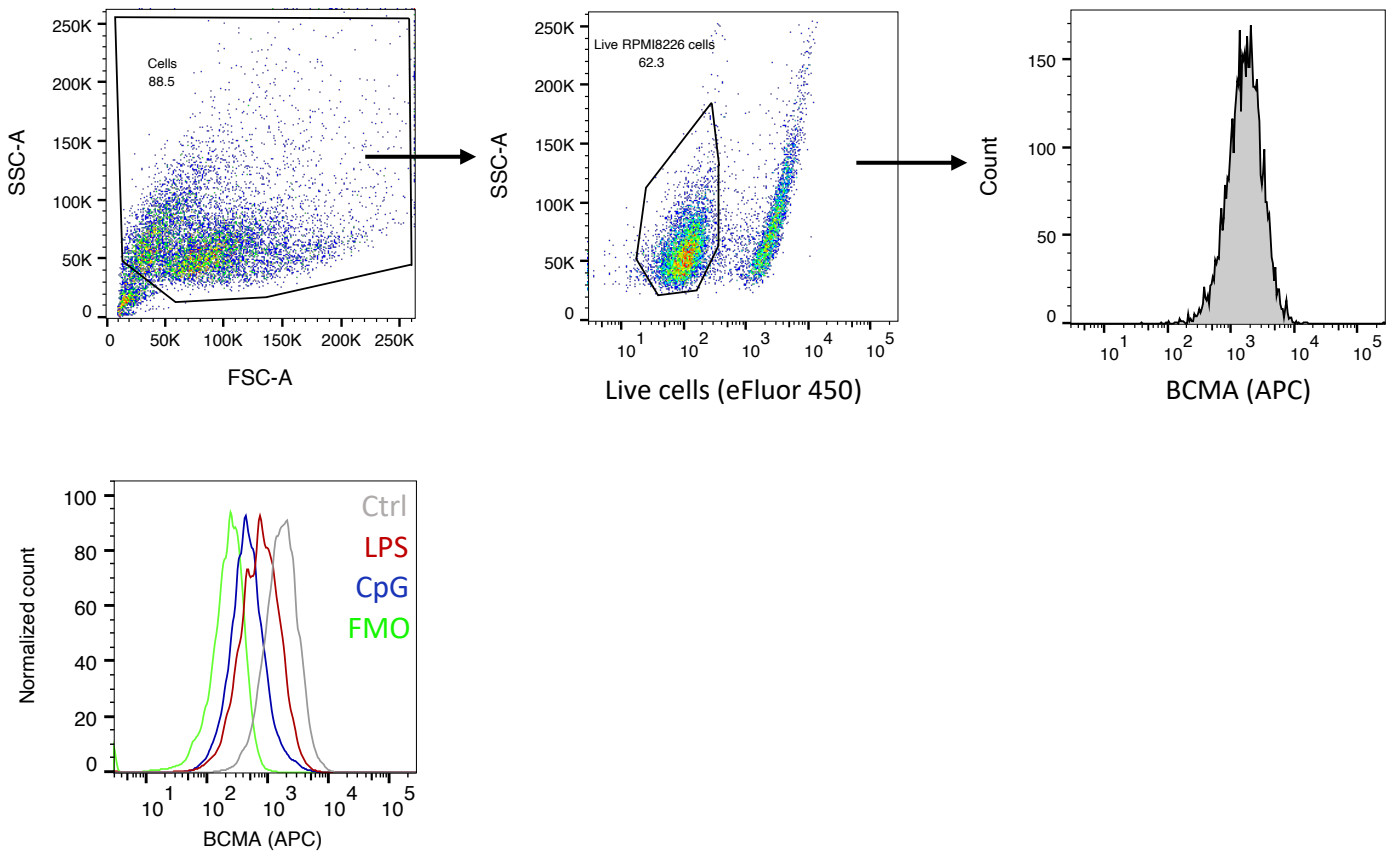

Supplementary Figure 4

Supplement: Supplementary Figure 1 — (A) Graphical presentation of extracellular acidification rate (ECAR) as a measure of glycolysis in RPMI-8226 cells treated with LPS (0.1 μg/mL) or CpG (1 μM) for 48 hours. The graph shows mean ± SD of more than 8 technical replicates from a representative experiment. Bar graph shows basal glycolysis (ECAR) ± SEM of 3 independent experiments. P-values were calculated using RM One-way ANOVA. *P≤ 0.05. (B) Graphical presentation of oxidative consumption rate (OCR) as a measure of oxidative phosphorylation in RPMI-8226 cells treated with LPS (0.1 μg/mL) or CpG (1 μM) for 48 hours. The graph shows mean ± SD of more than 8 technical replicates from a representative experiment. Bar graph shows basal respiration (OCR) ± SEM of 3 independent experiments. P-values were calculated using RM One-way ANOVA. *P≤ 0.05. (C) Relative mRNA expression of IRF4 and BCL2 was evaluated by RT-qPCR in RPMI-8266 cells treated with LPS (0.1 μg/mL) or CpG (1 μM). [file DataSheet_1.pdf]
