# Supplementary material for: Toll-like receptor signaling in multiple myeloma cells promotes the expression of pro-survival genes B-cell lymphoma 2 and MYC and modulates the expression of B-cell maturation antigen
Source: Front Immunol. 2024 Jun 7;15:1393906. doi: 10.3389/fimmu.2024.1393906 (PMC11190062; doi:10.3389/fimmu.2024.1393906)
Supplement: Supplementary file 2 [file Table_1.pdf]

Supplementary Table 1. Clinical and histopathological characteristics of MM patients. Samples were tested for the following cytogenetic abnormalities: t(4;14), t(11;14), t(14;16), t(14;20), 1q21+, del17p, del13q.

	**Age**	**Gender**	**Diagnosis/relapse sample**	**Cytogenetic abnormalities**
MM1	59	M	Relapse	t(4;14), 1q21+, del17p
MM2	65	M	Unknown	No deviation
MM3	55	M	Diagnosis	t(4;14), del17p
MM4	75	M	Unknown	1q21+
MM5	70	F	Unknown	t(14;16)
MM6	81	M	Diagnosis	1q21+
MM7	69	M	Diagnosis	No deviation
MM8	84	M	Diagnosis	No deviation
MM9	74	F	Diagnosis	t(11;14)
MM10	45	M	Diagnosis	No deviation
MM11	65	F	Diagnosis	No deviation
MM12	57	M	Diagnosis	1q21+
MM13	48	M	Diagnosis	1q21+
MM14	28	M	Diagnosis	No deviation
